# Video Games and Stress: How Stress Appraisals and Game Content Affect Cardiovascular and Emotion Outcomes

**DOI:** 10.3389/fpsyg.2019.00967

**Published:** 2019-05-07

**Authors:** Anne Marie Porter, Paula Goolkasian

**Affiliations:** Department of Psychological Science, The University of North Carolina at Charlotte, Charlotte, NC, United States

**Keywords:** stress, stress appraisal, cardiovascular – methods, video games, heart rate variabiity, blood pressure, emotion

## Abstract

Although previous studies have found that video games induce stress, studies have not typically measured all salient indicators of stress responses including stress appraisals, cardiovascular indicators, and emotion outcomes. The current study used the Biopsychosocial Model of Challenge and Threat (Blascovich and Tomaka, 1996) to determine if video games induce a cardiovascular stress response by comparing the effects of threat and challenge appraisals across two types of video games that have shown different cardiovascular outcomes. Participants received challenge or threat appraisal instructions, and played a fighting game (*Mortal Kombat*) or a puzzle game (*Tetris*). Study outcomes were heart rate variability, systolic and diastolic blood pressure, and positive and negative emotion ratings measured before, during and after gameplay. Results indicated that threat appraisal instructions increased negative emotion ratings and decreased heart rate variability, but not blood pressure, which is an essential marker for cardiovascular stress responses. Increased blood pressure and decreased heart rate variability was associated with fighting game players when compared with the puzzle game players, indicating a cardiovascular stress response; however, fighting game players also reported higher positive emotion ratings. Based on the study findings, video games do not induce stress responses like mental stressors used in previous research, demonstrating that the interactive player experience in video gaming may have more complex effects on stress outcomes. Future research should comprehensively measure biopsychosocial stress indicators and multiple emotional states over time to fully examine the relationship between video games and stress.

## Introduction

In recent years, research has demonstrated an interest in the effects of video games on stress. Some research studies have found that video games induce stress (Hasan et al., 2013; Hasan, 2015; Ferguson et al., 2016), while others have shown that video games reduce or manage stress (Reinecke, 2009; Russoniello et al., 2009; Roy and Ferguson, 2016). Mixed findings may be a result of two limitations in current video game studies. First, most research is not informed by established stress models that incorporate both cognitive and physiological factors, which has led to inconsistent conceptual definitions of stress across studies. Second, studies examining stress have used video games with different types of content, which can have different effects on stress outcomes. To address these limitations, the current study replicated methods used in previous stress research informed by the Biopsychosocial Model of Challenge and Threat (Blascovich and Tomaka, 1996), and compared stress outcomes across different types of video game content.

Stress is an important predictor of long term health. Chronic stress over time influences cardiovascular, metabolic, and immunological dysfunction, and contributes to the progression and exacerbation of multiple health problems including coronary heart disease, autoimmune disease, and diabetes (Harbuz et al., 2003; Pasquali et al., 2006; Steptoe and Kivimaki, 2012). Chronic stress is defined as exposure to a stressor persisting for several hours per day for weeks or months (Dhabar and McEwen, 1997). If video games have the potential to induce stress, regular video game players may be at-risk for chronic stress outcomes. In Entertainment Software Association (2018), 60% of Americans play video games daily, and the average online video gamer in America plays for 6.44 h per week (Limelight Networks, 2018). Certain populations such as pro-gamers play video games for 9.4 h per day (Han et al., 2012). The relationship between video games and chronic stress needs to be clearly understood for researchers and practitioners to predict health outcomes of players.

When encountering a stressor in the environment, the body activates a physiological stress response. During a stress response, the central, autonomic, endocrine, and immune regulatory systems interact to adapt to stressor demands and achieve physiological stability (Sterling and Eyer, 1988). As described in the General Adaptation Syndrome (Selye, 1956), stress responses occur within the sympathetic nervous system along the sympathetic-adrenal-medullary (SAM) axis and the hypothalamus-pituitary-adrenal (HPA) axis. The SAM axis stimulates catecholamine production in the adrenal medulla (e.g., adrenaline, noradrenaline, and epinephrine) and in parallel, the HPA axis stimulates the release of glucocorticoids (e.g., cortisol) in the adrenal cortex (Schoder et al., 2000; Gerra et al., 2001). Stress hormones signal the body to circulate more energy by influencing cardiovascular functioning. Acute mental stressors lead to increases in blood pressure and heart rate, and decreases in vagal tone and heart rate variability (Vieweg et al., 1997; Hjortskov et al., 2004). Chronic stress can result in these cardiovascular changes leading to health problems such as hypertension and coronary heart disease over time (McCrone et al., 2001; Steptoe and Kivimaki, 2012).

However, newer stress theories state that encountering a stressor does not always activate a physiological stress response, and the activation of a stress response is dependent on a cognitive process known as stress appraisal (Lazarus and Folkman, 1984). During stress appraisal, an individual determines whether a stressor is a challenge or threat to personal goals. If appraised as a threat, the stressor is considered an obstacle to personal goals and activates a stress response. If appraised as a challenge, the stressor is perceived to be a potential source of personal growth, and does not activate a stress response. Threat and challenge determinations are also influenced by an individual’s perceived ability to cope with a stressor based on the demand of the situation and the resources available (Blascovich and Tomaka, 1996). Situational demand is the potential required effort, uncertainty, and danger in dealing with the stressor, while available resources include both an individual’s internal and external resources such as knowledge, skills, self-efficacy, social support, or material support. If available resources are perceived as insufficient to deal with the stressor demands, then the stressor is appraised as a threat.

The Biopsychosocial Model of Challenge and Threat (Blascovich and Tomaka, 1996) added to previous stress theories by demonstrating how challenge and threat appraisals lead to different cardiovascular outcomes. Threat appraisals activate a physiological stress response by inducing the circulation of stress hormones from both the SAM axis and the HPA axis (Dienstbier, 1989). Challenge appraisals activate the SAM axis but not the HPA axis, and the body does not activate a physiological stress response. SAM activation with no corresponding HPA activation is considered an adaptive mobilization of energy resources that improves acute performance without the long-term negative effects on health (Seery, 2013). Studies have replicated these cardiovascular patterns of challenge and threat across several stress-inducing tasks including arithmetic problems, public speaking tasks, and social negotiations (Kelsey et al., 2000; Blascovich et al., 2004; Shimizu et al., 2011; Scheepers et al., 2012). In these studies, both challenge and threat appraisals showed increased heart rate, which indicates SAM activation; however, challenge appraisals decreased total peripheral resistance and had no effect on blood pressure, while threat appraisals increased total peripheral resistance and blood pressure (Tomaka et al., 1993, 1997, 1999; Tomaka and Blascovich, 1994; Blascovich et al., 1999; Mendes et al., 2001, 2002), indicating HPA activation.

The Biopsychosocial Model of Challenge and Threat (Blascovich and Tomaka, 1996) also describes how challenge and threat appraisals lead to differences in task performance and emotional states. Challenge appraisals have led to increased motor, cognitive, and social performance on a variety of tasks including military simulations, arithmetic tasks, standardized testing, simulated job interviews, and social negotiations (Larsson, 1989; Tomaka et al., 1993; Scheepers et al., 2012; Turner et al., 2012; Stout and Dasgupta, 2013). For emotion outcomes, threat appraisals induce negative emotions such as frustration or anxiety, and challenge appraisals induce positive emotions such as hope and excitement (Lazarus and Folkman, 1984). For example, participants who read threatening scripts before a public speaking task reported greater levels of anxiety post-task (Williams et al., 2010; Feldman et al., 2004), while participants who read challenging scripts before stressful tasks reported less anxiety and more self-confidence (Skinner and Brewer, 2002; Kaczmarek, 2009; Williams et al., 2010; Strack and Esteves, 2015).

Based on study findings with the Biopsychosocial Model of Challenge and Threat (Blascovich and Tomaka, 1996), threat and challenge appraisals lead to different physiological and emotional outcomes. Furthermore, the model demonstrates that both stress appraisals and cardiovascular outcomes need to be assessed within a study to make accurate conclusions about stress. Previous video game studies examining stress have not incorporated stress appraisals in the study design and have not measured all relevant cardiovascular indicators. For example, several studies demonstrated how playing video games increase heart rate, decrease heart rate variability and decrease cardiac coherence (Anderson, 2004; Bartlett et al., 2009; Hasan et al., 2013), but measuring cardiac indicators alone may not indicate a stress response. Increases in heart rate can reflect either challenge or threat appraisals, and unless HPA axis indicators such as blood pressure are also measured, we cannot determine if video games induce physiological stress (Blascovich and Tomaka, 1996).

In addition, previous video game studies have a limited focus on emotion. Studies with stress outcomes and violent video games have measured negative emotions such as anger, hostility, fear, and anxiety (Anderson, 2004; Ballard et al., 2006; Geslin et al., 2011), without assessing positive emotions or other potential emotional outcomes. Video games have a complex relationship with emotional processes, and video games are not unidimensional in their effects on emotional valence. The same video game can induce positive and negative cognitions and emotions simultaneously (Ravaja et al., 2008; Bosche, 2010). In addition, individuals can experience positive or negative emotions at different time points. Through appraisal processes, meta-emotion theories suggest that players can have positive emotional experiences of appreciation or enjoyment after playing games that induce negative emotions such as sadness or frustration (Oliver et al., 2015; Triberti, 2016). Reviews on emotion regulation have also demonstrated how video games can reduce negative emotions after feeling frustrated or stressed before or during gameplay (Villani et al., 2018).

The stress appraisal literature supports this need to measure a wider range of emotional states over time. According to Folkman and Lazarus (1985), distinct emotions are related to stress appraisal processes. Threat appraisal can induce feelings of anxiety, while challenge appraisal can induce feelings of excitement. When individuals appraise the potential harms and benefits of a stressful encounter, harm appraisals can induce feelings of frustration, while benefit appraisals can induce relaxation or happiness. The results of several studies demonstrated that appraisal-related emotions are dynamic and change over time during a stressful event, and different types of emotions may occur concurrently, such as excitement and anxiety (Folkman and Lazarus, 1985). Therefore, studies with video games and stress need to assess multiple types of emotion to capture more complex interactions between emotional states.

To add to the current literature, the current study manipulated stress appraisals before video gameplay and examined if cardiovascular and emotional outcomes as described in the Biopsychosocial Model of Challenge and Threat (Blascovich and Tomaka, 1996) can be replicated when playing a video game. Unlike previously tested stressors such as public speaking or exams (Blascovich et al., 1999; Strack and Esteves, 2015), video games contain elements of narrative, mechanics, and social context that influence user experiences (Elson et al., 2014), and it is important to examine if playing a video game may lead to different patterns in biopsychosocial stress outcomes. As in previous studies using the model, participants were given different instructions designed to induce a challenge or threat appraisal (Tomaka et al., 1997; Wang et al., 2016). Study outcomes were heart rate variability, blood pressure and emotion ratings related to threat, challenge, harm, and benefit appraisal processes.

We expect that threat and challenge instructions will result in decreased heart rate variability during and post-gameplay when compared to baseline, but the groups will differ in blood pressure changes. Participants who play under threat instructions are expected to show increased blood pressure while challenge instructions are not predicted to have an impact on blood pressure. Instruction groups are also predicted to differ in their emotion ratings. In comparison to challenge instructions, threat instructions should be associated with higher negative emotion ratings and lower positive emotion ratings post-gameplay.

Also of interest to the study is whether the emotional and cardiovascular changes associated with gameplay would be influenced by video game content. In a previous study, Bartlett and Rodeheffer (2009) compared video game content and found that a graphically realistic violent game (*Conflict Desert Storm*) increased heart rate while a non-violent game (*Hard Hitter Tennis*) decreased heart rate during gameplay. Other studies have demonstrated similar findings, and after playing video games involving fighting or shooting other human avatars such as *Mortal Kombat* or *Call of Duty*, participants have shown increases in heart rate, blood pressure, respiration rate, and decreases in heart rate variability (Panee and Ballard, 2002; Anderson, 2004; Bartlett et al., 2009; Hasan et al., 2013). Conversely, after playing puzzle games such as *Bejewled*, or non-realistic fighting games such as *LEGO: Marvel Superheroes*, players showed decreases in blood pressure, increases in heart-rate-variability, and slower respiration rates (Russoniello et al., 2009; Roy and Ferguson, 2016). Because video games differ in so many ways in addition to violent or non-violent content, it is difficult to attribute the difference in past research findings to any one factor. In spite of this, we compared two different games (*Mortal Kombat* and *Tetris*) that represent different kinds of game content to test whether they would influence outcome measures as suggested by past research or would interact with the threat and challenge appraisal instructions. In this way, we would be able to assess the degree to which the findings can be generalized across at least two kinds of video games or can be specialized within a specific kind of video game.

## Materials and Methods

### Participants

One hundred and forty-eight psychology students at The University of North Carolina at Charlotte participated in this study. Participant ages ranged from 18 to 38 (*M* = 19.92, *SD* = 3.18), and were 60% male and 60% White. All participants reported playing video games at least once before (including computer, console, and mobile games). On average, participants played games at least once a week (*M* = 2.25, *SD* = 1.19) for about 7 h a week (*M* = 7.76, *SD* = 7.84). Participants perceived themselves as moderately skilled at playing video games (*M* = 2.95, *SD* = 0.97). At baseline, participants showed normal levels of systolic blood pressure (*M* = 117.11, *SD* = 12.49) and diastolic blood pressure (*M* = 66.76, *SD* = 8.54). Only 2% of participants reported a previous diagnosis related to blood pressure outcomes including hypertension, diabetes, or renal disease. This study was carried out in accordance with the guidelines for human subjects in research and approved by The UNC Charlotte Institutional Review Board (IRB). All participants gave written informed consent in accordance with the Declaration of Helsinki, and received course credit for their participation.

### Materials

Video games were played on a Windows 10 PC with a Microsoft Xbox PC controller. Participants assigned to the fighting game condition played *Mortal Kombat: Komplete Edition*, which required players to fight in 10 one-on-one matches against computer opponents. Other versions of *Mortal Kombat* have increased heart rate and blood pressure in video game studies (Ballard et al., 2006; Bartlett et al., 2009), and was selected to examine how the Biopsychosocial Model of Challenge and Threat (Blascovich and Tomaka, 1996) applies to video gameplay. Participants assigned to the puzzle game condition played *Tetris Ultimate*, which required players to complete rows of falling shapes during a gameplay level, and participants restarted the level when the rows reached the top of the screen. Although previous studies have not examined the effect of playing *Tetris* on cardiovascular stress responses, *Tetris* was selected due to its ability to be placed on higher difficulty settings, unlike other puzzle games such as *Bejeweled.*

Higher difficulty settings were necessary for both video games to enable threat appraisals. Threat appraisals are influenced by perceived task demands (Lazarus and Folkman, 1984), and threat appraisals may not occur if participants play video games on easier difficulties. Matches in *Mortal Kombat* were set on “Hard,” and higher levels of difficulty increased the fighting skills of the computer opponent. *Tetris Ultimate* was set on difficulty level six, and higher levels of difficulty increased the speed in which shapes moved down the screen. Difficulty settings were chosen based on pre-testing with ten participants. In the selected difficulty settings, participants were able to complete at least one match in *Mortal Kombat* or five rows in *Tetris*, but could not complete all ten matches in *Mortal Kombat* or complete one continuous level of *Tetris* within the 15 min.

### Measures

#### Stress Appraisals

Manipulation checks for threat appraisals were conducted using self-report scales pre and post-gameplay. Stress appraisal scales were based on previous studies using the Biopsychosocial Model of Challenge and Threat (Tomaka et al., 1993; Mendes et al., 2001). Perceived threat was assessed by asking about situation-specific demands and participants’ self-concept of abilities; and scales were adapted to apply to video gameplay. Primary appraisal scales assessed the perceived demand of the video game, and secondary appraisal scales assessed participants’ perceived video game skills. Using seven point Likert scales, participants were asked “How demanding do you think the game will be (was)? (0 = not at all; 6 = extremely demanding)” and “Do you feel you have (had) the necessary skills to perform well in the game? (0 = not at all; 6 = definitely).” Higher scores on primary appraisal items and lower scores on secondary appraisal items indicated more threat appraisal.

#### Cardiovascular Outcomes

To indicate challenge and threat cardiovascular responses, heart rate variability and blood pressure were measured. Systolic and diastolic blood pressure were measured at baseline, before gameplay, and after gameplay using an Omron blood pressure monitor. Higher blood pressure indicated more sympathetic nervous system activity, which would occur in a cardiovascular stress response. Heart rate variability is an indicator of parasympathetic nervous system activity and vagal tone and measures the variability in the inter-beat intervals of the heart. Heart rate variability was measured continuously with a Polaris RS800CX heart rate monitor in 5-min intervals. Heart rate variability outcomes were compared using the square root of the mean square of successive R-R interval differences (RMSSD), which is a recommended time domain measure for short-term heart rate variability estimates (Task Force of the European Society of Cardiology and the North American Society of Pacing and Electrophysiology, 1996). Lower RMSSD scores indicated more sympathetic nervous activity and less parasympathetic nervous system activity, which would occur in a cardiovascular stress response. Data were analyzed using the ProTrainer 5 and Kubios programs, and artifacts were filtered using protocols within the Kubios program.

#### Emotion Ratings

Positive and negative emotion ratings were measured based on a previous study done by Folkman and Lazarus (1985), in which negative emotions were assessed in threat and harm-related emotion scales, and positive emotions were assessed in challenge and benefit-related emotion scales. All emotions were measured at baseline, pre-gameplay, and post-gameplay using a nine-point Likert scale (0 = not at all; 8 = extremely). To assess threat appraisal, participants rated the extent they felt “Worried,” “Fearful,” and “Anxious.” To assess challenge appraisal, participants rated the extent they felt “Determined,” “Confident,” and “Excited.” To assess harm appraisal, participants rated the extent they felt “Frustrated,” “Angry,” and “Disappointed.” To assess benefit appraisal, participants rated the extent they felt “Happy,” “Relaxed,” and “Proud.” Emotion scores were averaged across each sub-scale, and higher scores on each sub-scale indicated higher levels of that type of emotion. Emotion subscales had acceptable internal consistency for this sample at baseline, pre-gameplay, and post-gameplay. Cronbach alpha scores for all subscales ranged from 0.68 to 0.87.

#### Previous Video Game Experience

Previous video game experience was measured as a potential confound at baseline. Challenge and threat appraisals are influenced by perceived resources to cope with tasks demands, including previous experience or skills (Lazarus and Folkman, 1984). For example, participants who practiced a task prior to an evaluative performance were more likely to perceive the task as challenging (Blascovich et al., 1999). Several self-report questions assessed previous video game experience and were investigator-developed. Participants who had played games before (including PC, console, and cell phone games) indicated how often they play games (1 = Less than once a month; 4 = daily) and their perceived skill with video games in general (1 = not very skilled; 5 = extremely skilled). Participants who played weekly or daily indicated how many hours a week they play. Participants were also given a list of 13 video game genres, including “fighting” and “puzzle” games, and selected which video game genres they preferred to play.

#### Game Performance

Participant performance was assessed as a secondary measure of stress appraisal effects and was recorded during gameplay by the experimenter. For *Mortal Kombat* players, experimenters recorded the number of matches won out of ten. For *Tetris* players, experimenters recorded the highest number of points won during the level. Game performance scores were used to compare performance for threat and challenge appraisal groups within each of the game content conditions. Due to the use of different scoring metrics, performance was not compared between the two games.

#### Game Characteristics

After playing *Mortal Kombat* or *Tetris*, participants rated their game experience using an investigator developed, five-point Likert scale (1 = very little or not at all; 5 = very much). Participants rated the game on each of the following characteristics: violent, boring, enjoyable, and difficult.

#### Background Health Indicators

Several measures of physical health were collected at baseline to assess potential confounds for blood pressure outcomes. Body Mass Index was measured using a stadiometer and electronic scale, and participants were asked if they currently smoked, drank alcohol, and if they were previously diagnosed with hypertension, diabetes, or renal disease. Participants were also asked if they ingested caffeine within the last 4 h.

### Procedure

The current study used a factorial design with two between subject variables (stress appraisal and game content) and repeated measures at three time points: baseline, pre-gameplay, and post-gameplay. Participants were run individually in 1 h sessions, and randomly assigned to one of four conditions based on appraisal instructions and video game content. There were 37 participants per group. The four conditions were: challenge-fighting, threat-fighting, challenge-puzzle, and threat-puzzle. All four conditions were balanced by gender. At baseline, background video game experience and health indicator questionnaires were given to participants, and emotion ratings scales, heart rate variability, and blood pressure were measured. To measure heart rate variability continuously, participants wore a chest strap and *Polar* monitor. After sitting quietly for 5 min, three blood pressure readings were measured using an *Omron* cuff.

Video game instructions differed based on condition assignment and were developed from previous appraisal research (Tomaka et al., 1997; Wang et al., 2016). Participants in challenge appraisal conditions were instructed to win as many matches or get as many points as possible, and “think of the game as opportunity to overcome a challenge and succeed with continued effort.” Participants in threat appraisal conditions were given difficult performance-based instructions (win all 10 matches in *Mortal Kombat* or get 50,000 points in *Tetris Ultimate*), and were told they “would be evaluated based on their game performance and the speed with which they play the game.” After instructions, participants completed pre-gameplay primary and secondary stress appraisal ratings, emotion ratings, and two blood pressure readings. Participants were not given time to practice the controls before playing the video game.

Participants played the video game for 15 min and the experimenter recorded game performance (*Mortal Kombat* = number of matches won out of ten; *Tetris Ultimate* = highest number of points achieved within a single round). At 5 and 10 min of gameplay, the experimenter gave additional verbal prompts based on condition assignment. At 5 min, threat condition participants were told “You have 10 min remaining. You need to win matches/get points as fast as possible” and challenge condition participants were told “We want to see how you persist during a challenge, so don’t give up and win as many matches/get the highest score you can.” At 10 min, threat condition participants were told “You have 5 min remaining. You need to play faster and win more matches/get a higher score” and challenge condition participants were told “You’ll improve as you keep trying. Don’t give up.” Post-gameplay, participants completed stress appraisal ratings, emotion ratings, and game characteristic ratings (violent, boring, enjoyable, and difficult), and three final blood pressure readings were collected.

### Data Analysis

Data were analyzed using SPSS version 23. Study outcomes were examined separately using mixed analysis of variances *(ANOVAs)* to determine main and interaction effects of the two between-group variables: appraisal instructions (challenge or threat), and video game content (fighting or puzzle) with time as the repeated measures variable. Blood pressure readings, heart rate variability readings, and emotion ratings were analyzed at baseline, pre-gameplay, and post-gameplay. Heart rate variability changes were also continuously measured while playing the game and continuous readings were averaged and analyzed across 5-min intervals: baseline, before gameplay, 0–5 min of gameplay, 5–10 min, 10–15 min, and after gameplay. Blood pressure scores were averaged across two or three readings within 1-min intervals. *F*-tests reported for all within-group effects include the Greenhouse–Geisser correction when necessary to protect against possible violation of the sphericity assumption. *Post hoc* Bonferroni were used to examine main effects and simple main effects were analyzed to follow-up significant interaction effects. A significance level of 0.05 was used for all statistical tests.

## Results

### Participant Characteristics

Group differences in background video game experience, health indicators, and baseline measures were examined with a series of one-way *ANOVA*s or Chi-Square tests. The data showing no significant differences among the groups are presented in Tables 1, 2. An additional chi-square test was used to determine if participants preferred to play games within the genre they were assigned. Within the fighting game group, only 5% preferred to play fighting games, and within the puzzle game group, only 13% preferred to play puzzle games. There were no significant group differences based on puzzle game preference, χ^2^ (1, *N* = 148) = 0.98, *p* = 0.32, or fighting game preference, χ^2^ (1, *N* = 148) = 2.08, *p* = 0.15. Bivariate correlations for pre-gameplay and post-gameplay dependent variables are shown in Supplementary Tables S1, S2.

**Table 1:** Means (SD) for group comparisons across video game experience, health indicators, and baseline cardiovascular and emotion measures.

	Challenge-fighting	Threat-fighting	Challenge-puzzle	Threat-puzzle	*F*
How often play games	2.41 (1.21)	2.27 (1.24)	2.09 (1.09)	2.25 (1.23)	0.44
Hours/week play games	9.07 (9.42)	7.62 (7.95)	6.55 (7.71)	7.74 (6.05)	0.38
Perceived game skill	2.95 (1.13)	3.08 (1.04)	2.86 (0.67)	2.89 (0.99)	0.37
BMI	25.30 (4.82)	26.39 (9.08)	24.43 (5.14)	25.82 (5.23)	0.64
Threat emotions	1.55 (1.38)	2.03 (1.55)	2.13 (1.88)	1.94 (1.56)	0.93
Challenge emotions	4.66 (1.72)	4.77 (1.61)	4.65 (1.45)	3.89 (1.63)	2.36
Harm emotions	0.73 (1.48)	0.57 (1.10)	0.99 (1.75)	0.58 (0.82)	0.81
Benefit emotions	4.78 (1.71)	4.86 (1.57)	4.40 (1.71)	4.26 (1.29)	1.27
HRV	31.89 (19.74)	37.43 (22.77)	41.02 (29.99)	32.69 (19.09)	1.25
Systolic BP	116.77 (11.18)	118.26 (12.87)	118.28 (13.18)	115.14 (12.90)	0.53
Diastolic BP	67.17 (8.15)	66.39 (10.22)	67.99 (7.37)	65.48 (8.54)	0.58

*N = 148. BMI, Body Mass Index; HRV, Heart Rate Variability; BP, Blood Pressure.*

**Table 2:** Frequencies and percentages for group comparisons across health indicators.

Answered “Yes”	Challenge-fighting	Threat-fighting	Challenge-puzzle	Threat-puzzle	χ^2^
Currently smoke	3 (8%)	3 (8%)	6 (17%)	6 (17%)	2.47
Drink alcohol	16 (43%)	18 (49%)	20 (56%)	20 (56%)	3.77
Ingested caffeine 4 h before study	11 (30%)	15 (41%)	12 (33%)	8 (22%)	2.95
Previous blood pressure related diagnosis	0	2 (5%)	1 (3%)	0	3.69

*N = 148.*

### Manipulation Checks

#### Stress Appraisal Ratings

Mixed 2 × 2 *ANOVA*s were used to examine demand appraisals and skill appraisals at pre and post-gameplay. We predicted that threat appraisal instructions would result in higher demand appraisal ratings and lower skill appraisal ratings compared to challenge appraisal instructions. There was a significant main effect of challenge and threat instructions on demand appraisal ratings, *F*(1,144) = 12.35, *p* < 0.001, η_p_^2^ = 0.08, and skill appraisal ratings, *F*(1,144) = 17.56, *p* < 0.001, η_p_^2^ = 0.11. There were also significant interaction effects of appraisal instructions on demand appraisal ratings from pre to post-gameplay, *F*(1,144) = 3.88, *p* < 0.05, η_p_^2^ = 0.03, and skill appraisal ratings, *F*(1,144) = 17.56, *p* < 0.001, η_p_^2^ = 0.11. An analysis of simple main effects showed that threat appraisal groups had significantly higher demand ratings than challenge groups pre-gameplay, *F*(1,146) = 3.98, *p* < 0.05, and post-gameplay, *F*(1,146) = 15.91, *p* < 0.001. In addition, threat appraisal groups had significantly lower skill ratings post-gameplay, *F*(1,146) = 24.88, *p* < 0.001, but no significant differences pre-gameplay, *F*(1,146) = 1.37, *p* = 0.24. Compared to the challenge instruction groups, participants who received threat instructions believed the game was more demanding before and after the game, and they believed they were less skilled after playing the game. This indicated that the threat instructions worked as expected.

We also examined how game content influenced demand appraisals and skill appraisals. There was not a main effect of game content on demand appraisal, *F*(1,144) = 3.88, *p* = 0.18, or skill appraisal, *F*(1,144) = 0.34, *p* = 0.34, but there was a significant interaction effect of game content over time for skill appraisal, *F*(1,144) = 8.81, *p* < 0.01, η_p_^2^ = 0.06. An analysis of simple main effects showed that *Tetris* players had significantly higher skill appraisal ratings pre-gameplay, *F*(1,146) = 8.98, *p* < 0.01, indicating that they believed they would be more skilled at the game than *Mortal Kombat* players.

#### Game Characteristics

Game violence, boredom, enjoyment, and difficulty were compared across the four experimental groups using a series of one-way *ANOVAs*. There was a significant difference across groups for game violence ratings, boredom ratings, enjoyment ratings, and difficulty ratings (see Table 3). *Post hoc* Bonferroni comparisons were used to examine group differences. Both threat instruction groups rated the game as more difficult than the challenge groups, indicating that the threat instructions worked as expected. As expected, both *Mortal Kombat* groups rated the game as more violent than the *Tetris* groups, and there was not a significant difference in difficulty ratings between the games. Interestingly, *Mortal Kombat* groups also rated the game as less boring than *Tetris*, and participants who played *Mortal Kombat* with challenge instructions reported the highest level of enjoyment.

**Table 3:** Means (SD) of game characteristic ratings across study groups.

Game characteristic	Challenge-fighting	Threat-fighting	Challenge-puzzle	Threat-puzzle	*F*
Difficult	3.95 (0.88)	4.32 (0.78)	3.73 (0.96)	4.51 (0.51)	7.27^∗∗∗^
Violent	4.19 (0.84)	4.24 (1.06)	1.03 (0.16)	1.00 (0)	270.09^∗∗∗^
Boring	1.35 (0.72)	1.49 (0.69)	2.05 (1.05)	2.11 (1.24)	6.08^∗∗∗^
Enjoyable	3.97 (0.93)	3.43 (1.26)	3.22 (1.11)	2.89 (1.07)	7.27^∗∗∗^

*N = 148. ^∗∗∗^p < 0.001.*

### Heart Rate Variability Outcomes

**FIGURE 1:**
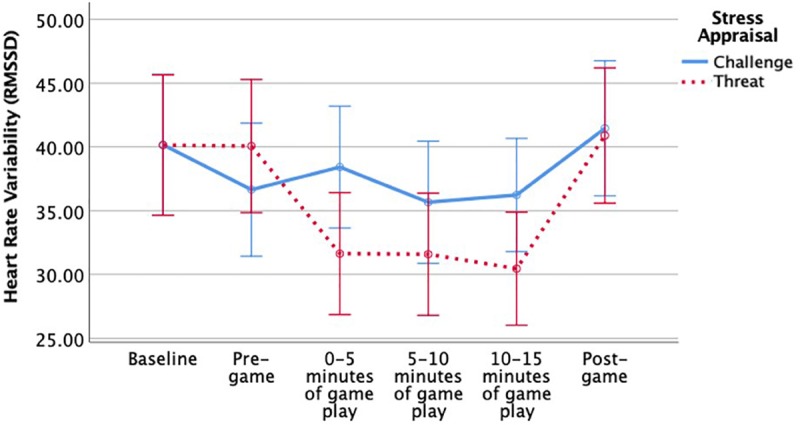
Significant interaction effect of stress appraisal instructions on heart rate variability over time. Error bars are 95% confidence intervals.

Changes in heart rate variability before, during and after game play were analyzed using a series of mixed *ANOVAs*. As predicted, heart rate variability as measured by RMSSD varied as a function of time in the experimental session (see Table 4). As shown in Figure 1, there was a significant instructional group by time interaction, *F*(3.25,468.37) = 7.46, *p* < 0.001, η_p_^2^ = 0.05; but there was no significant main effect of appraisal instructions, *F*(1,144) = 0.58, *p* = 0.45. An analysis of simple main effects showed that threat appraisal groups had significantly lower RMSSD than challenge groups during the first 5 min of gameplay, *F*(1,146) = 3.88, *p* < 0.05; but there were no other significant simple effects at other time points: pre-gameplay, *F* < 1, between 5 and 10 min of gameplay, *F*(1,146) = 1.40, *p* = 0.24, between 10 and 15 min of gameplay, *F*(1,146) = 3.23 *p* = 0.07, or post-gameplay, *F* < 1. In conclusion, participants who received threat appraisal instructions had more sympathetic activity during the start of the game, and Figure 1 shows that they had increased RMSSD after the game, indicating parasympathetic recovery.

**Table 4:** Changes in mean (SD) heart rate variability (RMSSD) before, during and after game play.

	Heart rate variability RMSSD
Baseline	35.76 (23.36)
Pre-gameplay	38.36 (22.68)
0–5 min during game	35.03 (21.16)
5–10 min during game	33.62 (21.01)
10–15 min during game	33.34 (19.71)
Post-gameplay	41.18 (22.96)
*F* (time)	19.10^∗∗∗^

*N = 148. RMSSD, Root mean square of the successive differences. ^∗∗∗^p < 0.001.*

**FIGURE 2:**
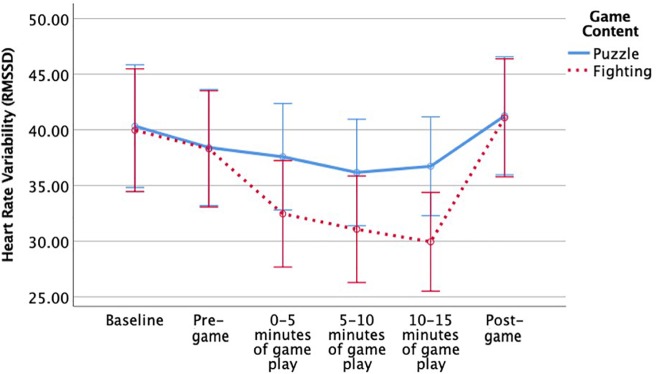
Significant interaction effect of game content on heart rate variability over time. Error bars are 95% confidence intervals.

There was not a significant main effect of game content on RMSSD, *F*(1,144) = 0.95, *p* = 0.33, but Figure 2 shows that changes in heart rate variability over time were specific to the kind of game, *F*(3.25,468.37) = 4.15, *p* < 0.01, η_p_^2^ = 0.03. An analysis of simple main effects showed that *Mortal Kombat* players had significantly lower RMSSD than *Tetris* players during the last 5 min of gameplay, *F*(1,146) = 4.49, *p* < 0.05. There were no significant simple main effects at other time points pre-gameplay, *F* < 1, between 0 and 5 min of gameplay, *F*(1,146) = 2.19, *p* = 0.14, between 5 and 10 min of gameplay, *F*(1,146) = 2.20 *p* = 0.14, or post-gameplay, *F* < 1. In conclusion, *Mortal Kombat* players had lower RMSSD during the last few minutes of the game, and Figure 2 shows that they had increased RMSSD after the game, indicating parasympathetic recovery. There was no significant appraisal × game content interaction for RMSSD, *F*(1,144) = 2.22, *p* = 0.14, or significant appraisal × game content interaction over time, *F* < 1.

### Blood Pressure Outcomes

Systolic and diastolic blood pressure also varied significantly over time in the experimental session. Table 5 presents the means (SD) at baseline, pre-gameplay, and post-gameplay for blood pressure and emotion outcomes. Although we predicted that threat appraisal instructions in comparison to challenge instructions would result in higher systolic and diastolic blood pressure post-gameplay, there were no main effects of appraisal instructions on systolic, *F*(1,144) = 0.07, *p* = 0.79, or diastolic blood pressure, *F*(1,144) = 1.12, *p* = 0.29. There were also no interaction effects of instructions over time on systolic, *F*(2,288) = 1.01, *p* = 0.36, or diastolic blood pressure, *F*(1.92,275.99) = 0.83, *p* = 0.43. Thus, appraisal instructions had no effect on blood pressure.

**Table 5:** Mean (SD) changes in blood pressure measures and emotion ratings over time.

	Baseline	Pre-gameplay	Post-gameplay	*F* (time)
Systolic BP	117.11 (12.49)	116.82 (12.85)	118.77 (13.08)	9.48^∗∗∗^
Diastolic BP	66.76 (8.54)	66.22 (8.56)	68.38 (9.12)	21.55^∗∗∗^
Threat emotions	1.91 (1.60)	2.25 (1.81)	1.57 (1.69)	14.05^∗∗∗^
Challenge emotions	4.49 (1.63)	4.64 (1.67)	4.09 (2.01)	8.53^∗∗∗^
Harm emotions	0.72 (1.33)	0.49 (0.94)	2.74 (2.16)	150.25^∗∗∗^
Benefit emotions	4.58 (1.58)	3.95 (1.66)	3.25 (1.74)	62.48^∗∗∗^

*N = 148. BP, Blood Pressure. ^∗∗∗^p < 0.001.*

There were no main effects of game content on systolic, *F*(1,144) = 1.64, *p* = 0.20, or diastolic blood pressure, *F*(1,144) = 0.78, *p* = 0.38, but there were interaction effects of game content over time on systolic, *F*(2,288) = 13.64, *p* < 0.001, η_p_^2^ = 0.09, and diastolic blood pressure, *F*(1.92,275.99) = 5.54, *p* < 0.01, η_p_^2^ = 0.04. An analysis of simple main effects showed that *Mortal Kombat* players had higher systolic blood pressure than *Tetris* players post-gameplay, *F*(1,146) = 6.74, *p* < 0.01. There were no significant simple main effects at baseline, *F* < 1 or pre-gameplay, *F* < 1.

**FIGURE 3:**
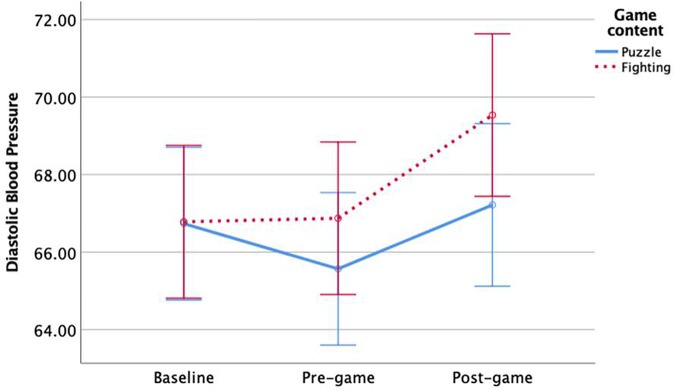
Significant interaction effect of game content on diastolic blood pressure over time. Error bars are 95% confidence intervals.

There were no significant simple main effects for diastolic blood pressure at baseline, *F* < 1, pre-gameplay, *F* < 1, or post-gameplay, *F*(1,146) = 2.41, *p* = 0.12. To investigate further, we examined the simple main effects of time when holding game content as a constant, and we found significant effects for both *Tetris*, *F*(2,145) = 7.25, *p* < 0.001, and *Mortal Kombat*, *F*(2,145) = 18.71, *p* < 0.001. As shown in Figure 3, both *Tetris* groups had a significant decrease in diastolic blood pressure after receiving instructions, and blood pressure significantly increased to baseline levels after the game. Both *Mortal Kombat* groups had a significant increase in blood pressure after the game. This indicates that *Mortal Kombat* players experienced a cardiovascular stress response while *Tetris* players did not.

There was no appraisal × game content interaction for systolic, *F*(1,144) = 1.07, *p* = 0.30, or diastolic blood pressure, *F* < 1. There was also no appraisal × game content interaction over time for systolic, *F* < 1, or diastolic blood pressure, *F* < 1.

### Emotion Outcomes

As indicated by the data presented in Table 5, there were significant changes in all emotional subscale ratings over time. For emotion outcomes, we predicted that threat appraisal instructions would result in higher threat emotions (anxious, worried, fearful) and harm emotion ratings (frustrated, angry, disappointed) post-gameplay, while challenge appraisal instructions would result in higher challenge (excited, determined, confident) and benefit emotion ratings (happy, relaxed, proud). There was a significant main effect of appraisal instructions on threat emotions, *F*(1,144) = 4.18, *p* < 0.05, η_p_^2^ = 0.03, challenge emotions, *F*(1,144) = 6.60, *p* < 0.01, η_p_^2^ = 0.04, and benefit emotions, *F*(1,144) = 5.43, *p* < 0.05, η_p_^2^ = 0.04. There were also significant interaction effects of appraisal instructions over time for all emotion subscales including threat emotions, *F*(2,288) = 3.24, *p* < 0.05, η_p_^2^ = 0.02, challenge emotions, *F*(1.58,227) = 6.44, *p* < 0.01, η_p_^2^ = 0.04, harm emotions, *F*(1.31,188.62) = 13.74, *p* < 0.001, η_p_^2^ = 0.09, and benefit emotions, *F*(1.70,244.27) = 10.39, *p* < 0.001, η_p_^2^ = 0.07.

An analysis of simple main effects showed that threat appraisal groups had significantly higher threat emotion ratings than challenge groups pre-gameplay, *F*(1,146) = 7.69, *p* < 0.01, but not post-gameplay, *F*(1,146) = 3.04, *p* = 0.08. Post-gameplay, threat appraisal groups reported significantly lower challenge emotion ratings, *F*(1,146) = 13.51, *p* < 0.001, higher harm emotion ratings post-gameplay, *F*(1,146) = 11.46, *p* < 0.001, and lower benefit emotions, *F*(1,146) = 16.61, *p* < 0.001. Pre-gameplay, there were no differences in challenge emotions, *F*(1,146) = 1.17, *p* = 0.28, harm emotions, *F* < 1, or benefit emotions, *F*(1,146) = 2.74, *p* = 0.10, and there were no differences between any emotion scales at baseline, *F* < 1. Overall, participants who received threat appraisal instructions reported more anxiety before the game, and reported more negative emotions and less positive emotions after the game.

There were significant main effects of game content on challenge emotions, *F*(1,144) = 6.50, *p* < 0.01, η_p_^2^ = 0.04, and benefit emotions, *F*(1,144) = 5.33, *p* < 0.05, η_p_^2^ = 0.04. There were also significant interaction effects of game content over time for challenge emotions, *F*(1.58,227) = 3.77, *p* < 0.05, η_p_^2^ = 0.03, and harm emotions, *F*(1.31,188.62) = 5.45, *p* < 0.01, η_p_^2^ = 0.04. An analysis of simple main effects showed that *Mortal Kombat* players had lower harm emotion ratings than *Tetris* players post-gameplay, *F*(1,146) = 6.00, *p* < 0.05, and higher challenge emotion ratings post-gameplay, *F*(1,146) = 10.16, *p* < 0.01. There were no significant differences in harm emotions at baseline, *F* < 1, or pre-gameplay, *F* < 1, or in challenge emotions at baseline, *F*(1,146) = 2.76, *p* = 0.10, or pre-gameplay, *F*(1,146) = 1.32, *p* = 0.25. Unexpectedly, *Mortal Kombat* players reported more positive emotions and less negative emotions than *Tetris* players after the game.

There was no appraisal × game content interactions for threat, *F* < 1, challenge, *F*(1,144) = 3.17, *p* = 0.08, harm, *F* < 1, or benefit emotions, *F* < 1. There were also no three-way interactions over time for threat, *F*(2,288) = 2.11, *p* = 0.12, challenge, *F* < 1, harm, *F*(1.31,188.62) = 1.37, *p* = 0.25, or benefit emotions, *F* < 1.

### Game Performance

Lastly, we predicted that threat instructions would result in lower game performance. Independent samples *t*-tests showed that there was neither a significant difference between challenge (*M* = 1.89, *SD* = 0.99) and threat instructions (*M* = 2.19, *SD* = 1.33) on the number of matches won in *Mortal Kombat*, *t*(72) = −1.09, *p* = 0.28, nor a significant difference between challenge (*M* = 12, 952.08, *SD* = 1,755.51) and threat instructions (*M* = 9,768.62, *SD* = 11, 363.61) on the highest number of points achieved in *Tetris*, *t*(71) = 1.24, *p* = 0.22. Appraisal instructions did not influence game performance.

## Discussion

The primary purpose of the current study was to determine if playing video games can induce stress by manipulating video game instructions to evoke threat or challenge appraisals. We predicted that participants who received threat instructions would experience a cardiovascular stress response (higher blood pressure and lower heart rate variability) and would report more negative emotions after the game. We predicted that participants who received challenge instructions would not experience a cardiovascular stress response and would report more positive emotions after the game.

We had mixed results in replicating previous findings using the Biopsychosocial Model of Challenge and Threat (Blascovich and Tomaka, 1996). As hypothesized, participants who received threat instructions reported more negative emotions such as frustration after gameplay, while participants who received challenge instructions reported more positive emotions such as excitement, happiness, or pride after gameplay. Previous studies using the Biopsychosocial Model of Challenge and Threat (Blascovich and Tomaka, 1996) have also found that challenge appraisals predict more positive and less negative emotions after a stress-inducing task (Skinner and Brewer, 2002; Williams et al., 2010; Strack and Esteves, 2015). Furthermore, participants who received threat instructions reported more anxiety before they started the game, indicating that threat instructions had the expected effect.

Cardiovascular outcomes were not consistent with previous findings using the model. Specifically, participants who received threat instructions had lower heart rate variability during the game as expected, but did not show increased blood pressure after gameplay. This contradicts previous research informed by the model, in which threat appraisals produced a cardiovascular stress response by increasing arousal and blood pressure, (Tomaka et al., 1993; Mendes et al., 2002; Turner et al., 2012; Gallagher et al., 2014). In the current study, the threat appraisal group had significantly higher arousal, indicating that threat instructions influenced cardiovascular outcomes; however, the lack of group differences in blood pressure indicates that threat appraisals did not induce a stress response when playing video games.

The current study was the first to examine the model with video gameplay, and our cardiovascular findings may differ from previous studies because video games do not share the same characteristics as other mental stressors. Previous studies using the model have examined situational stimuli such as public speaking, word searches, exams, and social interviews (Blascovich et al., 1999; Gaab et al., 2005; Mendes et al., 2008; Stout and Dasgupta, 2013; Strack and Esteves, 2015). To evoke stress appraisals, stressors must involve an aspect of social or self-evaluation related to task performance (Blascovich and Tomaka, 1996), and the former examples utilize social evaluation in the form of grades, performance assessment, or audience reactions. Video games, on the other hand, may not have the same context of social evaluation. Unlike an exam or public speaking performance, video games allow players to freely replay and practice scenarios without an evaluative audience, unless played in tournaments or other competitive settings. Video games also have beneficial effects on emotion regulation not found in other stressors. In studies with stressed individuals, playing video games have reduced negative emotions such as frustration, and participants have reported playing games for stress relief purposes (Colwell, 2007; Reinecke, 2009; Lobel et al., 2014). Although participants in the threat appraisal condition reported more negative emotions before and after the game, beneficial effects of emotion regulation during gameplay might have diminished the level of negative emotions and their effect on cardiovascular stress outcomes.

The secondary aim of the current study was to compare stress outcomes in the Biopsychosocial Model of Challenge and Threat (Blascovich and Tomaka, 1996) between a fighting video game (*Mortal Kombat*) and a puzzle video game (*Tetris*). There were significant differences in cardiovascular outcomes across different types of video games. For *Tetris* players, there were no changes in heart rate variability or blood pressure, but *Mortal Kombat* players had lower heart rate variability during gameplay and higher blood pressure after gameplay, indicating that *Mortal Kombat* players had a cardiovascular stress response. This supports previous video game studies in which similar fighting games increased arousal and blood pressure (Ballard and Wiest, 1996; Ballard et al., 2006). *Mortal Kombat* players also showed rapid cardiac recovery, and arousal returned to baseline levels within 5 min after gameplay. Rapid cardiac recovery has occurred in previous studies with other fighting video games, in which heart rate returned to baseline within 5–10 min after a 15-min gameplay session (Bartlett et al., 2009).

Previous studies suggest that the violent content within video games predict cardiovascular stress responses, and according to the General Aggression Model (Anderson and Bushman, 2002), aggressive cues in video games create internal aggressive states and increase arousal. However, this theory only partially explains study findings. Fighting game players in the current study did not report higher aggression-related emotions such as frustration or anger, but it is possible that aggressive cues within the fighting game may have contributed to threat appraisals and activated a cardiovascular stress response. It is uncertain which specific elements of violent games contributed to threat appraisals. One study suggests that graphic realism might play a role and found that graphically realistic shooting games increased arousal more than non-realistic shooting games (Bartlett and Rodeheffer, 2009). Another study showed that music might explain stress outcomes in violent video games. When playing a shooting game, participants who heard the in-game music had much higher cortisol levels after the video game than participants who did not hear music (Hébert et al., 2005). Therefore, it is unknown what specific features of *Mortal Kombat* contributed to cardiovascular stress responses.

Emotion findings differed when comparing fighting and puzzle games. After gameplay, *Mortal Kombat* players reported higher positive emotion ratings (excitement, happiness, and pride) and lower negative emotion ratings (frustration and anger) than *Tetris* players. These findings contradict previous research with video games, which have shown that puzzle games induce positive emotions such as relaxation (Russoniello et al., 2009) and fighting or shooting games induce negative emotions such as anger, hostility, or fear (Anderson, 2004; Bartlett et al., 2009; Geslin et al., 2011). Our findings could be explained by concepts within self-determination theory (Deci and Ryan, 2000). Individuals are motivated to play video games that satisfy psychological needs of autonomy, competence, and relatedness. Competence is the need for challenge and feelings of effectance during a task, and lower perceived competence has been related to more negative emotions such as frustration after gameplay (Przybylski et al., 2014).

In the current study, secondary stress appraisal ratings may indicate that puzzle game players felt less fulfillment of their competence needs. Before the video game, *Tetris* players believed they would be more skilled at the video game than *Mortal Kombat* players, and had a greater decrease in perceived skill after gameplay. Thus, *Tetris* players seemed to perform worse than expected, and experienced lower perceived competence and higher levels of frustration after gameplay. This explanation is also supported by enjoyment ratings post-gameplay. Oliver et al. (2015) found that higher feelings of competence were related to more enjoyment in video gaming. *Mortal Kombat* players rated the game as more enjoyable, which may indicate that they had more perceived competence. Our findings demonstrate that video games with violent content can induce positive emotions, and create more positive emotional experiences than non-violent games depending on psychological factors and gameplay context. Although previous studies have demonstrated how violent games may or may not induce negative emotions (Anderson, 2004; Ferguson and Rueda, 2010), very few studies have measured positive emotional outcomes. It is uncertain how positive emotional effects might influence cardiovascular stress for fighting game players over time. Studies have shown that positive emotions can be a protective factor and predict faster cardiovascular recovery after stressful events (Frederickson and Levenson, 1998) as well as healthier long-term HPA activity (Hoyt et al., 2015), which may buffer against potential negative cardiovascular effects of chronic stress.

### Limitations

The current study is limited by assessing HPA activation with systolic and diastolic blood pressure measurements. Although previous studies using the Biopsychosocial Model of Challenge and Threat (Blascovich and Tomaka, 1996) have used blood pressure as HPA indicators (Mendes et al., 2002), other studies have provided a more comprehensive examination of HPA activity by assessing cardiac output, total peripheral resistance, and salivary cortisol readings (Tomaka et al., 1999; Harvey et al., 2010). In addition, the current study conducted a cross-sectional experiment, and cannot make conclusions about the long-term effects of video gameplay and stress. Therefore, we cannot infer how playing fighting games over time could contribute to cardiovascular disease and other health outcomes.

The use of pre-existing video games limits our ability to examine how specific factors in fighting games influence stress. We were unable to compare performance across game content and could not control for the potential effects of in-game differences in design, narrative, and mechanics on physiological and emotional stress outcomes. For example, the selected games in the current study had different requirements for in-game success. *Mortal Kombat* players succeeded in the game by winning individual matches, while *Tetris* players succeeded by continuously receiving points during a single level. Different in-game performance experiences could have affected participants’ perceived post-game appraisal of competence and post-game emotion ratings across game conditions. Furthermore, the current study did not measure the differences in gameplay attempts upon losing a match or level, which may have also affected perceived competence and emotional states.

## Conclusion

The current study advanced video game research by applying the Biopsychosocial Model of Challenge and Threat (Blascovich and Tomaka, 1996) to video gameplay, and comprehensively measuring stress outcomes by assessing stress appraisals, cardiac activity, blood pressure, and emotion ratings. Although participants given stress-inducing instructions reported higher threat appraisals and higher levels of negative emotions, our study did not replicate previous findings with the model and video game players did not show a cardiovascular stress response in vascular measures. Our results highlight the necessity of assessing both cardiac and vascular indicators within a stress study, and future studies could include additional indicators of HPA activity such as total peripheral resistance and cortisol levels.

The type of video game had an effect on stress outcomes, and we found that fighting game players showed a cardiovascular stress response after gameplay while puzzle game players did not. However, fighting game players also reported higher levels of positive emotions after gameplay. Previous studies with violent video games have only focused on negative emotion outcomes such as aggression (Anderson, 2004), and violent video game research needs to also examine the role of positive emotions, which may have protective influences on stress. Future studies also need to examine longitudinal health outcomes of gaming populations that play more violent games on a weekly or daily basis, and determine if they are at-risk for long-term health issues related to chronic stress such as cardiovascular disease.

The application of the Biopsychosocial Model of Challenge and Threat (Blascovich and Tomaka, 1996) within the current study demonstrates how video games are a complex interactive experience, and produce different influences on stress outcomes compared to stressors tested in previous research. Unlike an arithmetic task or public speaking task, video games contain narratives, mechanics, music, player motivations, and other elements that have concurrent and unique influences on physiological systems and emotional states. Future research with video games should approach stress measurement with biopsychosocial methods to better understand the relationship between video gameplay and stress.

## Ethics Statement

This study was carried out in accordance with the recommendations of the Institutional Review Board committee and the Office of Research Compliance at The University of North Carolina at Charlotte with written informed consent from all subjects. All subjects gave written informed consent in accordance with the Declaration of Helsinki. The protocol was approved by the Institutional Review Board committee.

## Author Contributions

All authors who meet authorship criteria are listed, and all authors certify that they have participated sufficiently to take public responsibility for the content in the submitted work, including participation in the concept, design, analysis, writing, or revision of the manuscript. Furthermore, each author certifies that this work has not been and will not be submitted to or published in any other publication before its appearance in *Frontiers in Psychology*. AP contributed to the study design, data collection, data analysis, and drafting of the manuscript. PG contributed to the study design, data analysis, and revised the manuscript for important intellectual content. Both authors approved the final version of the manuscript for publication.

## Conflict of Interest Statement

The authors declare that the research was conducted in the absence of any commercial or financial relationships that could be construed as a potential conflict of interest.

## Abbreviations

ANOVA: Analysis of variance
HPA: Hypothalamus-pituitary-adrenal axis
RMSSD: Root mean square of the successive differences
SAM: Sympathetic-adrenal-medullary axis.
