# Supplementary material for: Video Games and Stress: How Stress Appraisals and Game Content Affect Cardiovascular and Emotion Outcomes
Source: Front Psychol. 2019 May 7;10:967. doi: 10.3389/fpsyg.2019.00967 (PMC6524699; doi:10.3389/fpsyg.2019.00967)
Supplement: Supplementary file 1 [file Table_1.docx]

| Table 1  *Bivariate correlations between dependent variables pre-gameplay* | | | | | | | | |
| --- | --- | --- | --- | --- | --- | --- | --- | --- |
| Variable | Demand appraisal | Skill appraisal | Threat emotions | Challenge emotions | Harm emotions | Benefit emotions | Systolic BP | Diastolic BP |
| Demand appraisal | - |  |  |  |  |  |  |  |
| Skill appraisal | -.33*** | - |  |  |  |  |  |  |
| Threat emotions | .38*** | -.17* | - |  |  |  |  |  |
| Challenge emotions | .11 | .44*** | .05 | - |  |  |  |  |
| Harm emotions | .12 | -.08 | .52*** | .09 | - |  |  |  |
| Benefit emotions | .01 | .33*** | -.25** | .63*** | .01 | - |  |  |
| Systolic BP | -.07 | .34*** | -.01 | .34*** | -.01 | .12 | - |  |
| Diastolic BP | .01 | .08 | .11 | .15 | .05 | .06 | .49*** | - |
| HRV | .09 | .02 | .04 | .08 | .03 | .04 | .20* | -.09 |

**Supplementary Materials**

*Note.* *indicates *p* < .05, **indicates *p* < .01, ***indicates *p* < .001; BP = Blood pressure, HRV = Heart rate variability.

| Table 2  *Bivariate correlations between dependent variables post-gameplay* | | | | | | | | |
| --- | --- | --- | --- | --- | --- | --- | --- | --- |
| Variable | Demand appraisal | Skill appraisal | Threat emotions | Challenge emotions | Harm emotions | Benefit emotions | Systolic BP | Diastolic BP |
| Demand appraisal | - |  |  |  |  |  |  |  |
| Skill appraisal | -.42*** | - |  |  |  |  |  |  |
| Threat emotions | .28** | -.26** | - |  |  |  |  |  |
| Challenge emotions | -.15 | .58*** | -.08 | - |  |  |  |  |
| Harm emotions | .32*** | -.46*** | .56*** | -.32*** | - |  |  |  |
| Benefit emotions | -.22** | .58*** | -.25** | .77*** | -.43*** | - |  |  |
| Systolic BP | .11 | .03 | -.08 | .26** | -.03 | .13 | - |  |
| Diastolic BP | .11 | .02 | .04 | .12 | .04 | .09 | .57*** | - |
| HRV | .04 | .04 | .05 | .12 | .03 | .01 | .21* | -.02 |

*Note.* * indicates *p* < .05, **indicates *p* < .01, ***indicates *p* < .001; BP = Blood pressure, HRV = Heart rate variability.
